# Invasive *Ageratina adenophora* can maintain its ecological advantages over time through releasing its autotoxicity by accumulating a bacterium *Bacillus cereus*

**DOI:** 10.1016/j.heliyon.2022.e12757

**Published:** 2022-12-30

**Authors:** Ai-Ping Wu, Zhong-Xi Bai, Jian Li, Hui Liu, Fa-Lin Chen, Man-Yun Zhang, Yan-Hong Wang, Mohamed Abdelaziz Balah, Ji-Hui Wen

**Affiliations:** a Ecology Department, College of Resources and Environment, Hunan Provincial Key Laboratory of Rural Ecosystem Health in Dongting Lake Area, Hunan Agricultural University, Changsha, 410128, China; b State Key Laboratory for Biology of Plant Diseases and Insect Pests, Institute of Plant Protection, Chinese Academy of Agricultural Sciences, Beijing, China; c College of Bioscience and Biotechnology, Hunan Agricultural University, Changsha, 410128, China; d School of Forestry and Bio-technology, Zhejiang Agriculture & Forestry University, Hangzhou, 311300, China; e Plant Protection Department,Ecology and Dry Lands Agriculture Division, Desert Research Center, 1 Mathaf El Matariya Street, El Matariya, 11753, Cairo, Egypt; f Institute of Plant Protection, Hunan Academy of Agricultural Sciences, Changsha, 410125, China

**Keywords:** Autotoxicity, Bacterium, Detoxification, Invasion, Advantage, Release, Decline, Over time

## Abstract

Plant invasive success is attributed to invaders’ ecological advantages over their native neighbors. However, increasing evidence suggests that these advantages are expected to attenuate over time because of natural enemy accumulation, ecological evolution of native species and autotoxicity. We determined how an invasive *Ageratina adenophora* could remain its competitive advantages over time by avoiding its autotoxicity. Our results highlighted that the autotoxicity of *A. adenophora* in its invaded soil was reduced by some microbes. Moreover, an autotoxic allelochemical, 2-coumaric acid glucoside, detected in the invaded soil, demonstrated distinctly autotoxic effects on its seed germination and seedling growth*.* However, the autotoxic effects were greatly alleviated by a bacterium *Bacillus cereus*, accumulated by *A. adenophora.* Furthermore, the allelochemical could be almost completely degraded by *B. cereus* within 96 h. Accordingly, we speculate that *A. adenophora* could aggregate *B. cereus* to release its autotoxicity maintaining its competitive advantages over time.

## 1 Introduction

More and more exotic species are intentionally or unintentionally introduced to new places because of the rapid progress of transportation and world trade, and some of them are becoming invasive species in their new communities, moreover, this trend is increasing throughout the world [1,2]. The invasive success of exotic species is often attributed to invaders' ecological advantages over their native competitors [3,4], while increasing evidence suggests that these advantages are expected to attenuate over time [3,[5], [6], [7]]. The advantage declines are usually caused by accumulating natural enemies or diseases, evolving improved competitive ability of native species and, of course, autotoxicity [[5], [6], [7], [8], [9]]. However, whether an invasive plant could remain its competitive advantages throughout its invasive stages by avoiding those declines are poorly documented. In this study, we only focus on the decline caused by autotoxicity, which is very common in invasive species [10,11]. Furthermore, autotoxicity may change the invaders' positive plant-soil feedback (PSF) into negative PSF, weakening invaders’ fitness and competitiveness [4,5,9]. However, the autotoxicity of invasive plants is not always taken into consideration when mechanisms are involved in maintaining the competitive ability of exotic species over time. Accordingly, studies of the mechanism by which an invasive species relieves its autotoxicity to remain its ecological advantages are scarce. We questioned how an invasive plant can maintain its competitive advantage over native competitors by relieving its autotoxic allelochemicals during its invasive stages.

Allelopathic (autotoxic) effects are largely affected by soil chemical, physical, and biotic conditions that determine the fate of allelochemicals in the environment [[12], [13], [14]]. It is well-known that native species from the origin of the invading species are not as sensitive as native species in recipient communities to allelochemicals excreted by some invasive plants [15]. This is because the native species in the recipient communities are usually considered more naïve to the new allelochemicals, to which the native species from the original source are adapted [15]. However, an increasing number of researchers have demonstrated that allelochemicals excreted by invasive plants are degraded by native soil biota, rather than native plants becoming adapted to the allelochemical [[16], [17], [18], [19], [20]]. For example [18], indicated that two main allelochemicals (oxo-10, 11-dehydroageraphorone (DTD) and 9b-hydroxyageraphorone (HHO)) of crofton weed were greatly alleviated or eliminated by soil biota in its invaded soil. Moreover [21], found that the degradation speed of DTD and HHO increased with increasing invasion history in the soil. However, no specific microorganisms and allelochemicals are clearly implied in their studies. Afterwards [22], suggested that DTD and HHO could be degraded by two strains (*Arthrobacter* sp. ZS and *Rhodococcus* sp. BS), which are purified from the soils invaded by crofton weed (*Ageratina adenophora*) and from habitats of native species. Then, the observation that specific microorganisms degrade specific allelochemicals excreted by invasive plants is firstly reported. Nonetheless, we do not know whether invasive plants can actively accumulate some microorganisms to relieve their autotoxicity, which actually declines their ecological advantages after a long time of local residence [9]. Therefore, it is very urgent to address this knowledge gap, and possible to support the positive PSF hypothesis in invasion ecology.

Crofton weed, *Ageratina adenophora* Sprengel (Synonym: *Eupatorium denophorum*), is a noxious invasive perennial forb originating from Mexico and Costa Rica, that has invaded more than 30 countries and regions in Asia, Africa, Europe, Oceania and the USA [23]. Since its introduction in the 1940s, crofton weed has invaded abandoned fields, agricultural fields, disturbed forests, orchards, pastures and roadsides in Southwestern China [24]. Moreover, it is spreading eastward and northward in China at a rate of 20 km per year by seed and vegetative propagation [24]. Its invasion causes a obvious decline of native species diversity, significant change of microbial communities, great loss of economy, and even faster mineral cycling [23–25]. Moreover, based on our field observation, the competitive advantages of crofton weed over native species are not reduced over its invasive time. Many water-soluble and liposoluble allelochemicals have been isolated and purified from crofton weed, and these allelochemicals are not only allelopathic to many native species but also autotoxic to itself [10,26]. Many experimental and observational results have showed that soil microbes might greatly alleviate the allelopathy of the invasive crofton weed directly or indirectly [21,22]. Unfortunately, the observation that specific soil microbe(s) degrade specific autotoxic allelochemicals is still short of direct evidence. Accordingly, the identity of microorganism(s) capable of degrading the autotoxic allelochemicals secreted by the invasive crofton weed and which autotoxic allelochemical(s) degraded by the microorganism(s) are still obscure and need further research, which is another focus of our study.

A type of allelopathic substance (2-coumaric acid glucoside) was found to be a large component of the allelochemicals isolated from the liposoluble composites from crofton weed, and pure 2-coumaric acid glucoside was used in this experiment [27]. We assessed whether crofton weed could accumulate some beneficial bacteria to detoxify the autotoxicity of 2-coumaric acid glucoside to keep its ecological advantages and facilitate its invasion success. The objectives in this study were to determine 1) whether the ecological advantage of crofton weed declined over its invasive time and whether this decline was associated with its autotoxicity; 2) whether crofton weed excreted the allelochemical 2-coumaric acid glucoside to the soil it invaded and caused autotoxic effects on itself; 3) which beneficial bacterium was accumulated by crofton weed, what the distribution pattern of the bacterium was in the soil and whether the aggregation of the beneficial bacterium was stimulated by the allelochemical; and 4) whether the bacterium could degrade the allelochemical and alleviate the autotoxic effects on crofton weed.

## 2 Materials and methods

### 2.1 Soil and seed collection

In June 2012, the soils were sampled from three different sites, invaded by *A. adenophora* for more than 40 years in Chengjiang County, Yuxi City [28]: a low land (24°55′33″N, 102°57′57″E, 1920 m a.s.l), a hillside (24°56′41″N, 102°56′39″E, 1944 m a.s.l) and a road side (24°50′5″N, 103°3′7″E, 1995 m a.s.l). At each site, based on the regimes of *A. adenophora*, four subcommunities were identified located at least 2000 m apart: a subcommunity with dominance of *A. adenophora* (Invaded), a subcommunity with *A. adenophora* and native species (Mixed), a subcommunity with only native species (Native) and a subcommunity with no plants (Bare). In each subcommunity, three 1.0 × 1.0 m^2^ plots were established located at least 20 m apart, and the vegetation and litter on the soil surface were removed. Five even soil samples from 0 to 10 cm in the plot were collected and blended together, treating them as a single composite sample per plot. Furthermore, ten kg of soil from the invaded, mixed and native subcommunities were also collected by the former method. After removing roots and gravel, all soil samples were air-dried at room temperature (25–33° Celsius), ground and homogenized by milling through a No. 40 mesh screen, and then stored at 4° Celsius for further analyses. Pure allelochemical substance (2-coumaric acid glucoside) was sincerely presented by professor Jian-Wen Tan from South China Botanic Garden, Chinese Academy of Sciences. Freshly mature seeds of *A. adenophora* were collected in 2012 from Yunnan province. All seeds were stored at 4° Celsius until use. Before germination, all seeds were surface sterilized with 5% hydrogen peroxide for 20 min, and then rinsed with distilled water several times.

### 2.2 Distribution pattern of Bacillus cereus

For each soil sample, 1.0 g of soil was added to 10 ml distilled water and shaken at 180 r/m, 25° Celsius for 20 min. Then, 1 ml of this solution was diluted 10 times twice by step, and 100 μl liquid was spread evenly into a 9-cm Petri dish with nutrient agar (NA) substrate (peptone 10 g, beef extract 3 g, NaCl 5 g, agar 15 g, water 1000 ml and pH 7.2–7.4) and incubated in a culture box at 28° Celsius in darkness, replicated three times, and each soil sample was also replicated three times. In this experiment, we used four soil types from three sites, three samples (plots) in each soil type from each site and nine replicates, resulting in a total of 324 dishes. After two days, bacteria were checked, and one dominant bacterium (>5% for mean value in the invaded soil samples) was isolated and one colony of this bacterium in a dish of the invaded soil sample was selected to culture to a single bacterium population. The pure bacterium was identified as Bacillus cereusby Personalbio Biological Technology Co., LTD, and this pure bacterium was kept for the following studies.

### 2.3 Effects of the allelochemical on the bacterium

The strain of *B. cereus* was added to 25 mm × 200 mm test-tube with 10 ml of four adopted sterile NA substrates (peptone 10 g, beef extract 3 g, NaCl 5 g, 1.0 (High), 0.3 (Middle), 0.1 (Low) mmol/L 2-coumaric acid glucoside and control-CK (sterile distilled water) 1000 ml and pH 7.2–7.4), then placed in an oscillating table shaking at 180 r/m, 25° Celsius for 24–48 h, and the number of the bacterium was counted under a microscope. Each treatment was replicated four times. In this experiment, we used four concentrations, two time treatments and four replicates, resulting in a total of 32 test-tubes.

### 2.4 Autotoxic and detoxification effects in solution

In our preliminary experiment, we had proved that the negative effects on seed germination of *A. adenophora* and other three native species were not caused by the 2-coumaric acid glucoside solution, and the negative effects were caused by the allelochemical itself (Supplementary Table 1). Furthermore, a previous study proved that 0.3 mmol/L of 2-coumaric acid glucoside showed evidently allelopathic (autotoxic) effects on some test species [27]. Accordingly, 0.3 mmol/L of the 2-coumaric acid glucoside was used in the following experiments to determine the autotoxic effects on *A. adenophora*. The strain of *B****.*** *cereus* was added to a 1000 ml triangular flask with 600 ml sterile distilled water and was cultured in an oscillating table as in experiment 2.3. The number of *B****.*** *cereus* was (6.4 ± 1.14) × 10^7^/ml after 24 h. The allelochemical concentration was set to 0.9 mmol/L for the following process. Four treatments were set in this experiment: control (3 ml sterile distilled water-CK), BC (2 ml sterile distilled water + 1 ml B***.*** *cereus* solution), BC + AS (1 ml sterile distilled water + 1 ml B***.*** *cereus* solution +1 ml allelochemical solution), and AS (2 ml sterile distilled water + 1 ml allelochemical solution). In each treatment, 30 seeds *A. adenophora* were arranged evenly in a separate Petri dish lined with two 5-cm pieces of filter paper, and 3 ml of due solution was added. Each treatment was replicated six times. In this experiment, we used four treatments and six replicates, resulting in a total of 24 dishes.

### 2.5 Autotoxic effects in soils with different invasive stages

In this experiment, 10 g of each invasive, mixed and native soils (sterilized and unsterilized) was added into a 5-cm Petri dish (approximately 3 cm in thickness). Thirty seeds of *A. adenophora* were buried evenly at a depth of 0.5 cm in each Petri dish, and each treatment was replicated six times. We used three soil types, two soil treatments and six replicates, resulting in a total of 36 dishes.

### 2.6 Autotoxic and detoxification effects in soil

In this experiment, only native soil (with no allelochemical 2-coumaric acid glucoside secreted by *A. adenophora*) was used and a total of 10 g soil from native communities was added to a 5-cm Petri dish (approximately 3 cm in thickness). The soil was treated with four solutions (CK, BC, BC + AS and AS) as experiment 2.4, and then kept at room temperature (23° Celsius-32° Celsius) for 24 h to react adequately. After that, 30 seeds were buried evenly at a depth of 0.5 cm in each Petri dish, and each treatment was replicated six times. In this experiment, we also used four treatments and six replicates, resulting in a total of 24 dishes.

For these culture experiments (experiments 2.4–2.6), all covered Petri dishes were incubated in culture boxes with 14 h of light (28° Celsius) and 10 h of darkness (20° Celsius) at greater than 85% relative humidity (similar to the climate of Yunan Province) for germination. Seed germination was recorded daily, and the seed was considered to be germinated when the radicle length was over 2 mm or rose up 2 mm through the soil surface. When no seeds had germinated for two continuous days, the experiments were terminated: 14 days in the soil experiment and 12 days in the solution experiment. Speed of germination was calculated referring to Ref. [29]. Seedlings germinated between the fourth and the seventh days were selected to measure their root length and shoot height. A total of 15 replicates per treatment was used.

### 2.7 Allelochemical degradation by the bacterium

In this experiment, two treatments were set to evaluate the allelochemical degradation ability of the isolated bacterium: control-CK (2 ml sterile distilled water + 1 ml 0.9 mmol/L allelochemical solution) and treatment -TR (1 ml sterile distilled water + 1 ml B*. cereus* solution +1 ml 0.9 mmol/L allelochemical solution). A total of 3 ml of due solution was added to each 5-cm Petri dish, and each treatment was replicated six times. All covered Petri dishes were placed randomly in culture boxes with 14 h of light (28° Celsius) and 10 h of darkness (20° Celsius) at greater than 85% relative humidity (similar to the climate of Yunan Province), and the allelochemical concentration of each dish was measured after 0, 12, 24, 48, and 96 h by a Shimadzu LC-20AT high-performance liquid chromatography (HPLC) (Shimadzu Corp., Japan) in South China Botanic Garden, Chinese Academy of Sciences, Guangzhou. The HPLC equipped with Agilent RP-18 (250 × 4.6 mm, 5 μm, Agilent, USA) fitted with the same guard column, detector SPD-M20A. A gradient of mobile phase composed of methanol (solvent A) and 0.5% acetonitrile (solvent B) was used according to the following program: starting with 90% A in 0.01 min and turning to 70, 80, 90 in 20, 23, 33 min and then stopping in 39 min with a flow rate of 1 ml/min at 230 nm. The injection volume was 20 μl. Each treatment was replicated six times. In this experiment, we used two solutions, five time treatments and six replicates, resulting in a total of 60 dishes.

### 2.8 Detection of 2-coumaric acid glucoside in soil

We collected and processed soil as in 2012 in the mixed subcommunities in the hillside site in June 2014. A total of 500 g of soil was diluted with 3 L of distilled water and stirred at 180 r/m, 25° Celsius for 24 h. The supernatant was filtered in vacuo and concentrated in a rotary evaporator until approximately three ml of solution was obtained. One milliliter of the solution was diluted to 10 ml by distilled water. Then, the diluted solution and 0.3 mmol/L 2-coumaric acid glucoside were detected by a Shimadzu LC-20AD HPLC (Shimadzu Corp., Japan) at Hunan Agriculture University, Changsha. The HPLC equipped with Inert Sustain C18 (250 × 4.6 mm, 5 μm, GL Science, Japan) fitted with the same guard column, detector SPD-20A and column oven CTO-10AS. The mobile phase and program were the same as in experiment 2.7.

### 2.9 Statistical analysis

To compare the number differences of *B****.*** *cereus* in all soil and plant parameters of seed germination and seedling growth that were measured in the four treatments, a one-way analysis of variance (ANOVA) followed by a Tukey HSD test was used. Additionally, the seed germination and seedling growth differences of crofton weed among the invasive, mixed and native soil with different sterilized treatments were analyzed by a two-way ANOVA followed by a Tukey HSD test. Similarly, number differences of *B****.*** *cereus* in the four solution treatments with different allelochemical concentrations between 24 h and 48 h were also compared by a two-way ANOVA, followed by a Tukey HSD test. Furthermore, concentration differences of the allelochemical measured at the five different periods (0 h, 12 h, 24 h, 48h, 96 h) in the allelochemical degradation experiment between CK and TR treatments were also compared by a two-way ANOVA, followed by a Tukey HSD test. Levene's test was used to test homogeneity of variances and treatments of the same level were compared by Post Hoc test with Duncan's test with the probability of error less than 5%. Statistical data analysis of the raw data was conducted using the software package R 3.5.2 [30].

Furthermore, to determine whether the bacterium *B. cereus* could degrade the allelochemical and facilitate the seed germination and seedling growth of the study species, the promotion effect (PE) on each measured indices of crofton weed promoted by the bacterium *B. cereus* was calculated as follows:

PE = (V_BC+AS_ - V_AS_) / V_AS_ × 100%

where V_BC+AS_ is the value of the treatment of BC + AS, and V_AS_ is the value of the treatment of AS. The values of PE can be positive or negative. If the value is positive, it means that the measured index is stimulated by *B. cereus* and if the value is negative, it means that the measured index is prohibited by *B. cereus* and the greater the value is, the bigger the promotion effect is.

## 3 Results

### 3.1 Autotoxicity effects in different soil

**Fig. 1:**
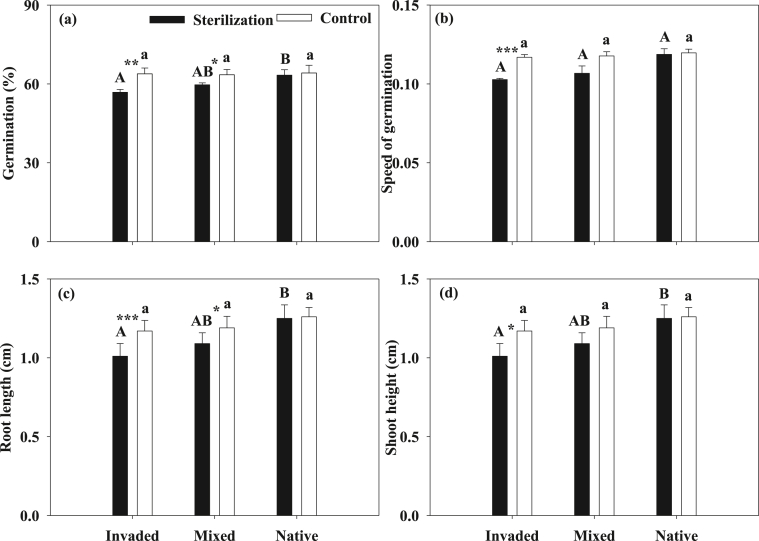
The effects on the seed germination and seedling growth of crofton weed by its invaded, mixed and native soil with sterilization or not, bars represent standard errors. Notes: different capital letters signify significantly different values among the different sterilized soil types, different low case letters signify significantly different values among the different unsterilized (control) soil types, and ‘*‘, ‘**’ and ‘***’ signifies significantly different values between the sterilized and unsterilized soil with the same soil type at P < 0.05, P < 0.01 and P < 0.001, respectively.

The results demonstrated that both the seed germination and seedling growth of *A. adenophora* were not significantly different among the invasive, mixed and native unsterilized soil (Fig. 1, ANOVA, *p* > 0.05). However, the inhibition strength of seed germination and seedling growth of *A. adenophora* were decreased from the invasive, mixed to native sterilized soil except the speed of germination (Fig. 1, ANOVA, *p* < 0.05). Furthermore, the seed germination and seedling growth of *A. adenophora* were significantly suppressed in the sterilized soil compared to unsterilized soil, especially for the invaded soil (Fig. 1 and Table 1). Moreover, the negative effects on the seed germination and seedling growth of *A. adenophora* distinctly reduced from the invaded, mixed to the native soil (Fig. 1) (see Table 1).

**Table 1:** ANOVA results of the seed germination and seedling growth of crofton weed in the invaded, mixed and native soil with sterilization or not, P < 0.05 are in bold.

Source	Germination			Germination speed			Root length			Shoot height		
	df	F	P	df	F	P	df	F	P	df	F	P
Soil type (ST)	2	4.589	**0.014**	2	6.799	**0.002**	2	2.296	0.118	2	4.150	**0.026**
Sterilization (S)	1	8.354	**0.006**	1	5.770	**0.020**	1	8.713	**0.006**	1	9.872	**0.004**
ST × S	2	3.025	0.057	2	1.354	0.267	2	1.826	0.178	2	2.064	0.145
Residual	84			84			84			84

**Table 2:** The promotion effects on the seed germination and seedling growth of crofton weed caused by the bacterium *B****.****cereus*.

Indices	Solution (%)	Soil (%)
Germination	61.84 ± 7.26	11.01 ± 0.86
Speed of germination	93.34 ± 12.17	9.67 ± 0.58
Root length	82.99 ± 10.65	211.53 ± 14.24
Shoot height	137.33 ± 10.32	4.47 ± 0.23

### 3.2 Bacterium distribution and promotion

**Fig. 2:**
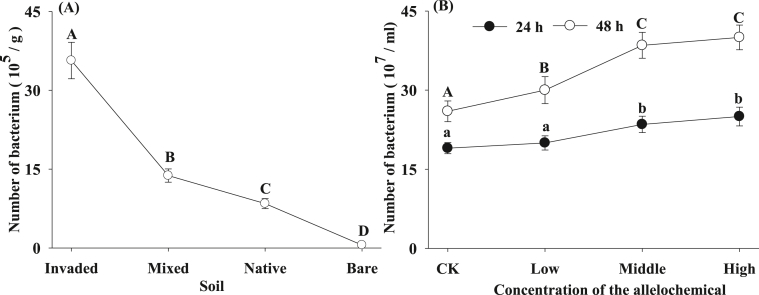
The number of *B***.***cereus* in the invaded, mixed, native and bare soil (A) and in culture solution (B) with distilled water (CK) and Low, middle and high concentrations of 2-coumaric acid glucoside in 24 and 48 h. Different letters means significantly different (ANOVA, p < 0.01), bars represent standard errors.

The number of *B****.*** *cereus* in the soil decreased sharply with the increased distance from the invaded subcommunity of *A. adenophora* to the bare soil (Fig. 2, ANOVA, *p* < 0.01), namely, the number of *B****.*** *cereus* was highest in the soil of the invaded subcommunity, intermediate in the soil of mixed and native subcommunities, and lowest in the bare soil. Furthermore, the growth of *B****.*** *cereus* was promoted by the allelochemical 2-coumaric acid glucoside (Fig. 2, ANOVA, *p* < 0.01).

### 3.3 Release of autotoxicity

**Fig. 3:**
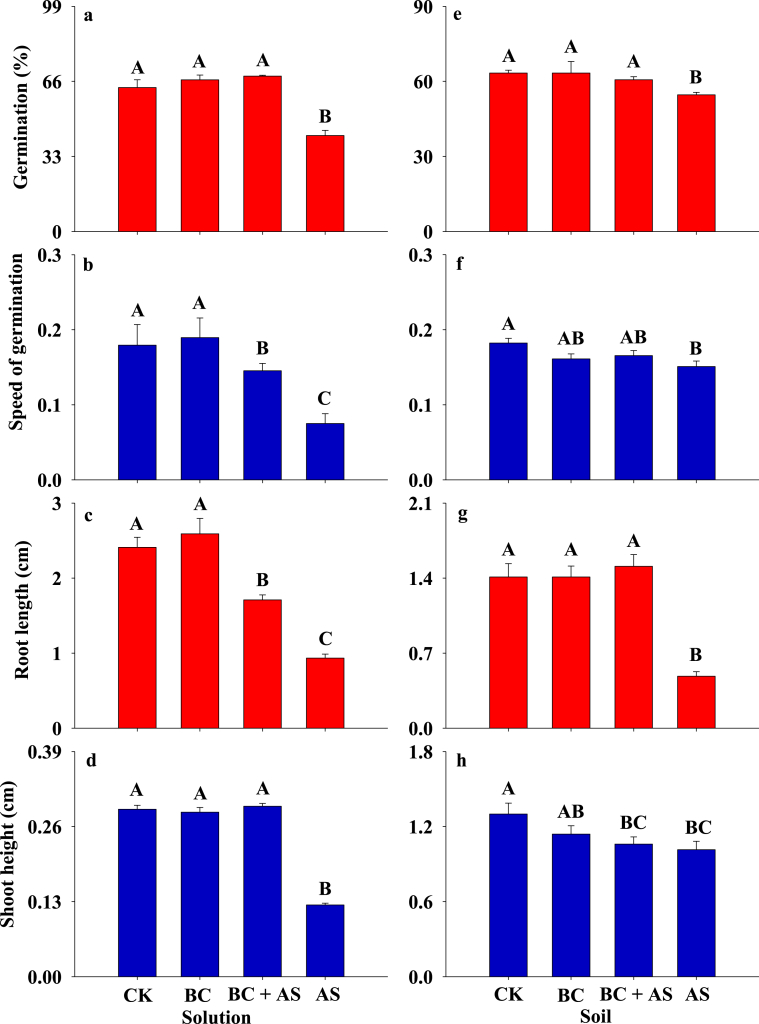
The seed germination and seedling growth of crofton weed under four treatments (control (3 ml sterile distilled water-CK), BC (2 ml sterile distilled water + 1 ml B**.***cereus* solution), BC + AS (1 ml sterile distilled water + 1 ml B**.***cereus* solution +1 ml allelochemical solution), and AS (2 ml sterile distilled water + 1 ml allelochemical solution)) in the solution and soil experiments, different letters represent significantly different (P < 0.05; ANOVA), bars represent standard errors.

The results showed that the allelochemical 2-coumaric acid glucoside could be detected in the soil invaded by crofton weed (Fig. 3). Moreover, the results demonstrated that the allelochemical 2-coumaric acid glucoside evidently prohibited seed germination and seedling growth of the target species in both the solution and the soil experiments (Fig. 3, ANOVA, *p* < 0.05). However, the autotoxic impacts on seed germination and seedling growth of the target species were apparently relieved by the bacterium in both the solution and the soil experiments (Table 2), although only speed of germination and shoot height of *A. adenophora* in soil experiment were both not different between the treatments of BC + AS and AS (Fig. 3, ANOVA, *p* > 0.05).

### 3.4 Allelochemical degradation and detection

**Fig. 4:**
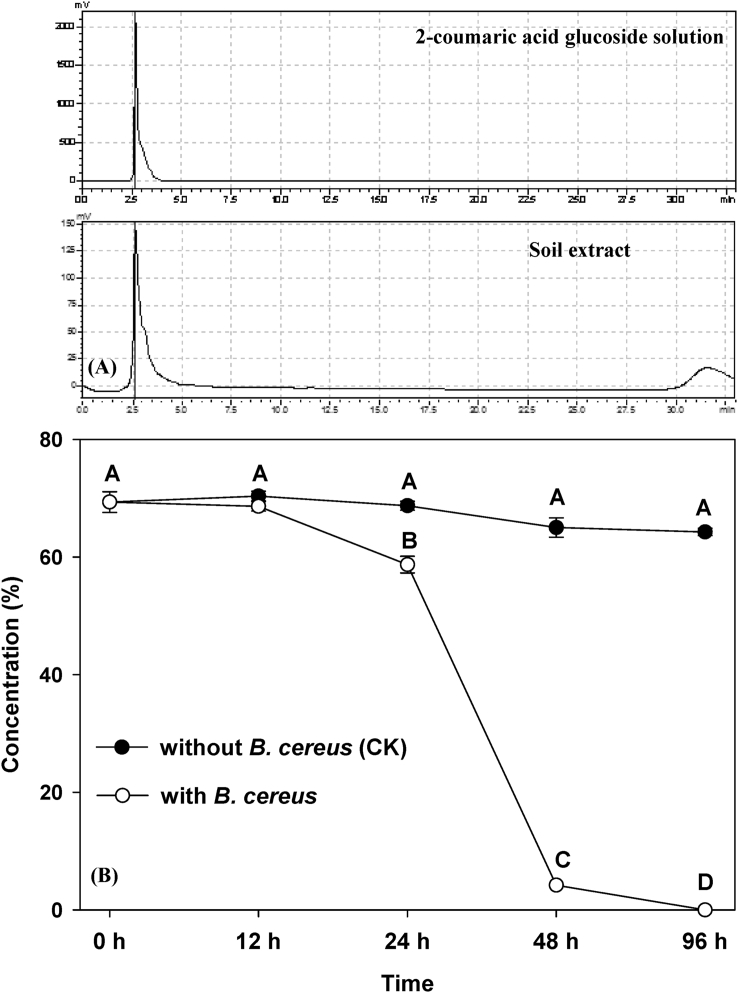
The absorbance of 2-coumaric acid glucoside in the soil extract (A) detected by HPLC and the concentration of 2-coumaric acid glucoside in solution without *B***.***cereus* (CK) and with *B***.***cereus* (B), different letters represent significantly different (P < 0.05; ANOVA), bars represent standard errors.

In our experiment, the allelochemical 2-coumaric acid glucoside could be detected in the soil invaded by invasive crofton weed with the help of HPLC (Fig. 4-A). Furthermore, the allelochemical could be degraded promptly within 96 h by the bacterium *B****.*** *cereus* after being determined by HPLC analysis (Fig. 4-B and supplementary Figure S1).

## 4 Discussion

### 4.1 Bacterium accumulation

As observed in our experiment, invasion of crofton weed might significantly alter the abundance of the bacterium, *B****.*** *cereus*. (Fig. 2-A). Furthermore, *B****.*** *cereus* can be accumulated by crofton weed in the soil of the invaded community (Fig. 2-A). The results demonstrated that the growth and reproduction of the bacterium were stimulated by 2-coumaric acid glucoside (Fig. 2-B). These results agree well with our third hypothesis that the bacterium was stimulated by the allelochemical. Moreover, the allelochemical 2-coumaric acid glucoside was also detected in the soil invaded by crofton weed through HPLC analysis (Fig. 4-A), indicating that the allelochemical was secreted to the soil by crofton weed, which is consistent with our second hypothesis that crofton weed could excrete the allelochemical 2-coumaric acid glucoside to the soil it invaded. Accordingly, we can presume that crofton weed may produce the allelochemical for protection, and the bacteria is able to consume and break down the allelochemical thus accumulating in the soil surrounding the plant invader. Although other allelochemicals and rich nutrients in the soil invaded by crofton weed could also aggregate microorganisms [31,32], it is obvious that *B****.*** *cereus* is induced to accumulate around crofton weed by 2-coumaric acid glucoside based on our results (Figs. 2–4). Similarly, some soil rare bacterium and nitrogen-fixing bacterium are reported to accumulate around the root of the invasive *A. adenophora* [33,34]. Previous research shows that the soil under crofton weed is usually more fertile (such as higher organic carbon, nitrogen and phosphoros contents) than the soil in native communities [35]. Moreover, soil fertility is considered to affect microbial community structure much more than plant species [36]. Accordingly, we can speculate that both soil fertility and 2-coumaric acid glucoside might promote the aggregation of *B****.*** *cereus* around crofton weed.

### 4.2 Degradation of the allelochemical

The allelopathic impacts of invasive species alleviated by native soil microorganisms in the invaded range have been directly [22] or indirectly [16–21] observed in previous studies. However, the observation that invasive plants can actively accumulate some microorganisms to relieve their autotoxicity has not been documented to our best knowledge. In this study, we demonstrated that an explicit allelochemical (2-coumaric acid glucoside) secreted by the invasive crofton weed was degraded and the autotoxicity of this allelochemical was greatly alleviated, by a soil explicit microbe (*B****.*** *cereus*) within several days in our study (Fig. 4 and supplementary Figure S1), which addresses this knowledge gap. Moreover, the results are correspondent with our fourth hypothesis that the bacterium could degrade the allelochemical and alleviate the autotoxic effects on crofton weed. Accordingly, the reason that crofton weed escapes from its autotoxin of 2-coumaric acid glucoside and gains a competitive advantage over its native competitors is largely attributed to its ability to aggregate *B****.*** *cereus* to release the autotoxicity rather than its adaptation to the allelochemicals [16,17]. Similarly, some invasive species can use their allelochemicals to attract some harmful fungi to attack their native competitors to gain their invasive advantages, namely, positive PSF [31,37]. Furthermore, the autotoxic effect was found to be alleviated by some soil microorganisms in some native species [38]. Moreover, some invasive species including *A. adenophora* is reported to accumulate some beneficial bacterium and fungi around its roots (Chen et al., 2109; [34,39,40]. Taken together, we can speculate that the invasive crofton weed can accumulate a bacterium, *B****.*** *cereus*, to alleviate its autotoxic allelochemical, which could weaken its negative PSF and benefit its competitive advantages. To our knowledge, we are the first to report this phenomenon. This special ability could promote the invasive success of crofton weed.

### 4.3 Maintain competitive advantages by relieving autotoxicity

Our results clearly highlighted that seed germination and seedling growth of crofton weed were greatly inhibited by the allelochemical both in the solution and soil experiments (Fig. 4). Furthermore, the allelochemical 2-coumaric acid glucoside could be detected in its invaded communities (Fig. 4-A). These results suggest that 2-coumaric acid glucoside could be autotoxic to crofton weed itself, which is well agreed with our second hypothesis that 2-coumaric acid glucoside was excreted to the soil by crofton weed and showed autotoxic effects on itself. The autotoxic effect agrees well with the effects of other allelochemicals isolated from crofton weed [10], indicating that the autotoxicity of crofton weed is very common. Actually, the autotoxicity might be unfavorable to the maintenance of its ecological advantages (or the invasion success of an invasive plant), although it is well known that the allelochemicals excreted by the invasive plants usually negatively impact the native species in the recipient communities [15].

However, our results demonstrated that the autotoxic effects of crofton weed increased over its invasive time only in the sterilized soil but not in the unsterilized soil (Fig. 1). These results strongly suggest that the ecological advantage declines of crofton weed caused by its own autotoxicity might be greatly reduced by some microbes in the soil (Fig. 1). However, which micro-organism (s) play the function of relieving the autotoxicity of the crofton weed to keep its competitive advantages over native species during its invasive stages and successfully invades the recipient communities remains unknown. As discussed above, our results highlight that invasive crofton weed can aggregate a beneficial bacterium *B. cereus* around itself to alleviate the autotoxic allechemical, 2-coumaric acid glucoside, exerted by itself and detected in the invaded soil. Thus, the results imply that it is the bacterium *B. cereus* that relieves the autotoxicity (at leat for the autotoxic allechemical, 2-coumaric acid glucoside) of crofton weed in the invaded soil (Figs. 1–4). Furthermore, the results highlight that the autotoxicity reduction of crofton weed increased with its invasion time, which is associated with the number of aggregated bacterium *B. cereus* (Figs. 1–2). Our results are well agreed with [21]; who found that the degradation speed of DTD and HHO increased with increasing invasion history in the soil. Moreover, these results imply that the ecological advantage declines of some invasive plants could occur in a short residence time (more than 40 years in our study), which is in accordance with the findings of [41]; although the ecological advantages of most invasive plants may decline after 100–300 years of local residence [4,5]. Taken together, the above results highlight that crofton weed could maintain its ecological advantages by avoiding the autotoxicity of 2-coumaric acid glucoside in this research, although the autoxicity might be caused by many other allechenicals or their interactions [12–14]. Surely, if an invasive species could keep its competitive advantages by reducing the effects of its autotoxicity in its long-time invaded habitats, the invasive success of this species would be more possible than that of other invasive species that cannot reduce their autotoxicity. Accordingly, we can presume that crofton weed has the ability to maintain its ecological advantages in its long-time invaded communities through alleviating the effects of its autotoxicity caused by its own allelochemical 2-coumaric acid glucoside by accumulating the beneficial bacterium *B****.*** *cereus*. Thus, this ability confers crofton weed much more competitive and successfully invasive than those have not this ability.

## 5 Conclusions

Our results highlight that the invasive crofton weed accumulates *B****.*** *cereus* to alleviate the autotoxic allelopathic effects on its own seed germination and seedling growth and keeps its ecological advantages over its native competitors during its invasive stages, thus facilitating its invasion success.

## Author contribution statement

APW, YHW and MAB conceived and designed the experiments; APW, ZXB, HL, JL and FLC performed the experiments; ZXB, MYZ, JHW and MAB analyzed and interpreted the data; APW, YHW and ZXB contributed reagents, materials, analysis tools or data; APW, YHW, ZXB and JHW wrote the paper.

### Funding statement

Professor Aiping Wu was supported by 10.13039/501100004735Natural Science Foundation of Hunan Province [2018JJ2162].

### Data availability statement

Data will be made available on request.

## Declaration of competing interest

The authors declare that they have no known competing financial interests or personal relationships that could have appeared to influence the work reported in this paper.

## References

[1] Delavaux C.S., Weigelt P., Dawson W., Duchicela J., Essl F., van Kleunen M. (2019). Mycorrhizal fungi influence global plant biogeography. Nat Ecol Evol.

[2] Hulme P.E. (2009). Trade, transport and trouble: managing invasive species pathways in an era of globalization. J. Appl. Ecol..

[3] Dostál P., Müllerová J., Pyšek P., Pergl J., Klinerová T. (2013). The impact of an invasive plant changes over time. Ecol. Lett..

[4] Warren R.J., Candeias M., Labatore A., Olejniczak M., Yang L. (2019). Multiple mechanisms in woodland plant species invasion. J. Plant Ecol..

[5] Diez J.M., Dickie I., Edwards G., Hulme P.E., Sullivan J.J., Duncan R.P. (2010). Negative soil feedbacks accumulate over time for non-native plant species. Ecol. Lett..

[6] Stricker K.B., Harmon P.F., Goss E.M., Clay K., Flory S.L. (2016). Emergence and accumulation of novel pathogens suppress an invasive species. Ecol. Lett..

[7] Blossey B., Nuzzo V., Dávalos A., Mayer M., Dunbar R., Landis D.A. (2021). Residence time determines invasiveness and performance of garlic mustard (*Alliaria petiolata*) in North America. Ecol. Lett..

[8] Hawkes C.V. (2007). Are invaders moving targets? The generality and persistence of advantages in size, reproduction, and enemy release in invasive plant species with time since introduction. Am. Nat..

[9] Van de Voorde T.F.J., Ruijten M., van der Putten W.H., Bezemer T.M. (2012). Can the negative plant–soil feedback of *Jacobaea vulgaris* be explained by autotoxicity?. Basic Appl. Ecol..

[10] Zhu X.Z., Guo J., Shao H., Yang G.Q. (2014). Effects of allelochemicals from *Ageratina adenophora* (Spreng.) on its own autotoxicity. Allelopathy J..

[11] Gala-Czekaj D., Dziurka M., Bocianowski J., Synowiec A. (2022). Autoallelopathic potential of aqueous extracts from Canadian goldenrod (*Solidago canadensis* L.) and giant goldenrod (*S. gigantea* Aiton). Acta Physiol. Plant..

[12] Inderjit, Wardle D.A., Karban R., Callaway R.M. (2011). The ecosystem and evolutionary contexts of allelopathy. Trends Ecol. Evol..

[13] Ehlers B.K., Berg M.P., Staudt M., Holmstrup M., Glasius M., Ellers J. (2020). Plant secondary compounds in soil and their role in belowground species interactions. Trends Ecol. Evol..

[14] Hierro J.L., Callaway R.M. (2021). The ecological importance of allelopathy. Annu. Rev. Ecol. Evol. Syst..

[15] Callaway R.M., Thelen G.C., Rodriguez A., Holben W.E. (2004). Soil biota and exotic plant invasion. Nature.

[16] He W.M., Feng Y.L., Ridenour W.M., Thelen G.C., Pollock J.L. (2009). Novel weapons and invasion: biogeographic differences in the competitive effects of *Centaurea maculosa* and its root exudate (+/-)-catechin. Oecologia.

[17] Thorpe A.S., Thelen G.C., Diaconu A., Callaway R.M. (2009). Root exudate is allelopathic in invaded community but not in native community: field evidence for the novel weapons hypothesis. J. Ecol..

[18] Zhu X., Zhang J., Ma K. (2011). Soil biota reduce allelopathic effects of the invasive *Eupatorium adenophorum*. PLoS One.

[19] Dawson W., Schrama M. (2016). Identifying the role of soil microbes in plant invasions. J. Ecol..

[20] Fahey C., Flory S.L. (2021). Soil microbes alter competition between native and invasive plants. J. Ecol..

[21] Li Y.P., Feng Y.L., Chen Y.J., Tian Y.H. (2015). Soil microbes alleviate allelopathy of invasive plants. Sci. Bull..

[22] Li Y.P., Feng Y.L., Kang Z.L., Zheng Y.L., Zhang J.L., Chen Y.J. (2017). Changes in soil microbial communities due to biological invasions can reduce allelopathic effects. J. Appl. Ecol..

[23] Poudel A.S., Jha P.K., Shrestha B.B., Muniappan R. (2019). Biology and management of the invasive weed *Ageratina adenophora* (Asteraceae): current state of knowledge and future research needs. Weed Res..

[24] Wang R., Wang J.F., Qiu Z.J., Meng B., Wan F.H., Wang Y.Z. (2011). Multiple mechanisms underlie rapid expansion of an invasive alien plant. New Phytol..

[25] Feng Y.L., Lei Y.B., Wang R.F., Callaway R.M., Valiente-Banuet A., Inderjit (2009). Evolutionary tradeoffs for nitrogen allocation to photosynthesis versus cell walls in an invasive plant. Proc. Natl. Acad. Sci. USA.

[26] Liu H.M., Zhou L.J. (2019). Plant autotoxicity review - 1. Families: Acanthaceae to campanulaceae. Allelopathy J..

[27] Zhang M., Bi H.H., Zhou Z.Y., Ren H., Tan J.W., Wan F.H. (2011). Isolation and identification of a potential allelochemical from the invasive plant *Eupatorium adenophorum*. J biosafety.

[28] Wu A.P., Liu L., Qi L., Zhong W., Liang Y., Chen F. (2019). Rapid nitrogen and phosphorus homeostasis transformation in *Eupatorium adenophorum* during invasion. Weed Res..

[29] Wardle D.A., Ahmed M., Nicholson K.S. (1991). Allelopathic influence of nodding thistle (*Carduus nutans* L.) seeds on germination and radicle growth of pasture plants. New Zeal J Agr Res.

[30] R Core Team (2018).

[31] Eppinga M.B., Rietkerk M., Dekker S.C., De Ruiter P.C., Van der Putten W.H. (2006). Accumulation of local pathogens: a new hypothesis to explain exotic plant invasions. Oikos.

[32] Liu W.X., Niu H.B., Wan F.H., Liu B. (2010). Effects of leachates of the invasive plant; *Ageratina adenophora* (Sprengel) on soil microbial community. Acta Ecol. Sin..

[33] Chen L., Fang, Zhou J., Yang Z.P., Dong X.F., Dai G.H. (2019). Enrichment of soil rare bacteria in root by an invasive plant *Ageratina adenophora*. Sci. Total Environ..

[34] Fang K., Bao Z.S.N., Chen L., Zhou J., Yang Z.P., Dong X.F. (2019). Growth-promoting characteristics of potential nitrogen-fixing bacteria in the root of an invasive plant *Ageratina adenophora*. PeerJ.

[35] Jiang Z.L., Liu W.X., Wan F.H., Li Z.Y. (2008). Effects of *Ageratina adenophora* (Spreng) invasion on soil nutrient properties and their seasonal dynamics. J Agro-Environm Sci.

[36] Kao-Kniffin J., Balser T.C. (2008). Soil fertility and the impact of exotic invasion on microbial communities in Hawaiian forests. Microb. Ecol..

[37] Mangla S., Inderjit, Callaway R.M. (2008). Exotic invasive plant accumulates native soil pathogens which inhibit native plants. J. Ecol..

[38] Džafić E., Pongrac P., Likar M., Regvar M., Vogel-Mikuš K. (2013). The arbuscular mycorrhizal fungus *Glomus mosseae* alleviates autotoxic effects in maize (*Zea mays* L.). Eur. J. Soil Biol..

[39] Borda V., Longo S., Marro N., Urcelay C. (2021). The global invader *Ligustrum lucidum* accumulates beneficial arbuscular mycorrhizal fungi in a novel range. Plant Ecol..

[40] Sun Y.Y., Zhang Q.X., Zhao Y.P., Diao Y.H., Gui F.R., Yang G.Q. (2021). Beneficial rhizobacterium provides positive plant–soil feedback effects to *Ageratina adenophora*. J. Integr. Agric..

[41] Cunard C.E., Lankau R.A. (2017). Declining survival across invasion history for *Microstegium vimineum*. PLoS One.

