# Supplementary material for: Invasive Ageratina adenophora can maintain its ecological advantages over time through releasing its autotoxicity by accumulating a bacterium Bacillus cereus
Source: Heliyon. 2022 Dec 30;9(1):e12757. doi: 10.1016/j.heliyon.2022.e12757 (PMC9849935; doi:10.1016/j.heliyon.2022.e12757)
Supplement: Supplementary file 1 — Multimedia component 1 [file mmc1.doc]

Supplementary Figure 1

Supplementary Table 1

| Species | Control (%) | Treatment (%) |
| --- | --- | --- |
| *Ageratina adenophora* | 67.33±3.42^A^ | 66.67±2.14^A^ |
| *Medicago sativa* | 86.67±2.04^A^ | 88.33±2.08^A^ |
| *Poa annua* | 82.35±2.65^A^ | 81.53±1.76^A^ |
| *Eupatorium lindleyanum* | 58.33±0.38^A^ | 58.33±2.23^A^ |
